# A novel locus conferring resistance to *Puccinia hordei* maps to the genomic region corresponding to *Rph14* on barley chromosome 2HS

**DOI:** 10.3389/fpls.2022.980870

**Published:** 2022-10-06

**Authors:** Mehnaz Mehnaz, Peter M. Dracatos, Hoan X. Dinh, Kerrie Forrest, Matthew N. Rouse, Robert F. Park, Davinder Singh

**Affiliations:** ^1^School of Life and Environmental Sciences, Plant Breeding Institute, University of Sydney, Sydney, NSW, Australia; ^2^Department of Animal, Plant and Soil Sciences, La Trobe University, AgriBio, Bundoora, VIC, Australia; ^3^Agriculture Victoria, AgriBio, Centre for AgriBioscience, Bundoora, VIC, Australia; ^4^USDA-ARS Cereal Disease Laboratory, Department of Plant Pathology, University of Minnesota, St. Paul, MN, United States

**Keywords:** characterization, genetic resistance, KASP, NLR, *RphAGG396*

## Abstract

Barley leaf rust (BLR), caused by *Puccinia hordei,* is best controlled through genetic resistance. An efficient resistance breeding program prioritizes the need to identify, characterize, and map new sources of resistance as well as understanding the effectiveness, structure, and function of resistance genes. In this study, three mapping populations were developed by crossing Israelian barley lines “AGG-396,” “AGG-397,” and “AGG-403” (carrying unknown leaf rust resistance) with a susceptible variety “Gus” to characterize and map resistance. Genetic analysis of phenotypic data from rust testing F_3_s with a *P. hordei* pathotype 5457 P+ revealed monogenic inheritance in all three populations. Targeted genotyping-by-sequencing of the three populations detected marker trait associations in the same genomic region on the short arm of chromosome 2H between 39 and 57 Mb (AGG-396/Gus), 44 and 64 Mb (AGG-397/Gus), and 31 and 58 Mb (AGG-403/Gus), suggesting that the resistance in all three lines is likely conferred by the same locus (tentatively designated *RphAGG396*). Two Kompetitive allele-specific PCR (KASP) markers, HvGBSv2-902 and HvGBSv2-932, defined a genetic distance of 3.8 cM proximal and 7.1 cM distal to *RphAGG396*, respectively. To increase the marker density at the *RphAGG396* locus, 75 CAPS markers were designed between two flanking markers. Integration of marker data resulted in the identification of two critical recombinants and mapping *RphAGG396* between markers- Mloc-28 (40.75 Mb) and Mloc-41 (41.92 Mb) narrowing the physical window to 1.17 Mb based on the Morex v2.0 reference genome assembly. To enhance map resolution, 600 F_2_s were genotyped with markers- Mloc-28 and Mloc-41 and nine recombinants were identified, placing the gene at a genetic distance of 0.5 and 0.2 cM between the two markers, respectively. Two annotated NLR (nucleotide-binding domain leucine-rich repeat) genes (r2.2HG0093020 and r2.2HG0093030) were identified as the best candidates for *RphAGG396*. A closely linked marker was developed for *RphAGG396* that can be used for marker-assisted selection.

## Introduction

Cultivated barley, *Hordeum vulgare*, is considered as a founder crop of modern agriculture (Park et al., 2015) ranking fourth in terms of global production after maize, rice, and wheat. Barley can be affected by four rust diseases: leaf rust, crown rust, stripe rust, and stem rust. Among these, barley leaf rust (BLR; also known as brown rust), caused by the fungus *Puccinia hordei* Otth, is one of the most destructive and widely distributed worldwide. The disease has caused yield losses documented at up to 32% in Australia and North America, with losses in very susceptible varieties even reported as being as high as 60% (Cotterill et al., 1992; Castro et al., 2012; Park et al., 2015). BLR is best controlled through deployment of resistant cultivars (Singh et al., 2015; Rehman et al., 2017; Sardar et al., 2022) and therefore breeding for leaf rust resistance is one of the prime objectives of many barley breeding programs worldwide.

To date, 28 *Rph* (resistance to *Puccinia hordei*) genes have been cataloged and mapped in barley (Mehnaz et al., 2021a), comprising 25 seedling or all stage resistance (ASR) genes (*Rph1*-*Rph19*, *Rph21*-*Rph22*, and *Rph25*-*Rph28*) and three adult plant resistance (APR) genes-*Rph20*, *Rph23*, and *Rph24* (Hickey et al., 2011; Singh et al., 2015; Ziems et al., 2017). Most of the cataloged ASR genes have limited value in breeding because they have been rendered ineffective by pathotypes of *P. hordei* with matching virulence (Park et al., 2015) or due to possible associated linkage drag (Bishnoi et al., 2022) in the case of those derived from wild species (*H. spontaneum* or *H. bulbosum*). To enhance the genetic base and durability of effective resistance in combating epidemics caused by new leaf rust pathotypes, there is a need to identify, characterize and map new resistance genes as well as understand their effectiveness, structure, and function (Park et al., 2015; Li et al., 2016; Wani et al., 2022).

Mapping the genetic basis of disease resistance requires the development of populations segregating for disease resistance response, phenotypic accuracy, marker genotyping, and subsequent trait association analysis to locate the target locus (Hamwieh et al., 2018). Population size, structure, and the type and density of molecular markers employed for mapping play vital roles in determining the resolution of the maps generated (Richards et al., 2017; Tomar et al., 2022). A variety of molecular markers have been developed over the last 30 years and used in mapping traits of interest in crops. For example, in barley, *Rph13* (Jost et al., 2020), *Rph26* (Yu et al., 2018), and *Rph27* (Rothwell et al., 2020) have been mapped/fine-mapped in bi-parental mapping populations using SNPs (single nucleotide polymorphisms), CAPS (cleaved amplified polymorphic sequences) and HRM (High resolution melting), and indels (insertion–deletion events), respectively. Gene mapping provides the foundation for map-based cloning, which ultimately helps in understanding the structure and function of genes (Wani et al., 2022). Markers identified through gene mapping can be used in marker-assisted selection (MAS) to increase plant breeding efficiency as well in gene pyramiding to attain durable resistance.

In the current study, three bi-parental mapping populations were developed to undertake inheritance studies and the identification of genomic regions conferring resistance to BLR. Three barley genotypes (AGG-396, AGG-397, and AGG-403) were selected from a Middle Eastern and Central Asian barley germplasm collection based on their resistance to all predominant Australian pathotypes of *P. hordei* (Mehnaz et al., 2021b). We conducted the present studies with three objectives: (i) to identify the genomic regions associated with resistance to *P. hordei* in genotypes AGG-396, AGG-397 and AGG-403; (ii) to fine map the resistance (tentatively designated as *RphAGG396*); and (iii) to develop closely linked markers for MAS of *RphAGG396.*

## Materials and methods

### Plant and pathogen material

Three barley lines [AGG-396 (AUS#403467), AGG-397 (AUS#403468) and AGG-403 (AUS#403774)] from Israel were investigated in this study. The lines were obtained from Australian Grains Genebank (AGG) Horsham, Victoria. Mapping populations were developed by crossing each of the three lines with a leaf rust susceptible (S) genotype “Gus.” F_1_s (first filial generation) derived from the three crosses were grown, selfed, and advanced to the F_2_ generation. The F_2s_ were sown as space planted long rows in the field and single plants were harvested as F_3_ families (AGG-396/Gus = 105; AGG-397/Gus = 120; AGG-403/Gus = 126 families). A single seed from each F_3_ family was then planted in a 20 cm pot @ three lines per pot to advance to the F_4_ generation. Sixteen pathotypes of *P. hordei* (14 of Australian origin and two of North American origin; Table 1) were used according to the objectives of the corresponding experiments.

**Table 1:** *Puccinia hordei* pathotypes used in this study, along with origin and their virulence profile.

Pathotypes	Origin	Culture^#^	Virulence profile^**^
200 P−^*^	Australia	518	*Rph8*
201 P+^*^	Australia	480	*Rph1*, *Rph8, Rph19,* and *Rph25*
210 P+^*^	Australia	482	*Rph4*, *Rph8, Rph14, Rph19,* and *Rph25*
220 P+^*^	Australia	485	*Rph5, Rph8, Rph19,* and *Rph25*
220 P + 13^*^	Australia	577	*Rph5*, *Rph8, Rph13*, and *Rph19*
242 P+	Australia	531	*Rph2*, *Rph6*, *Rph8,* and *Rph19*
253 P−	Australia	490	*Rph1, Rph2, Rph4, Rph6,* and *Rph8*
5652 P+	Australia	561	*Rph2*, *Rph4, Rph6, Rph8, Rph9, Rph10, Rph12, Rph13,* and *Rph19*
5610 P+^*^	Australia	637	*Rph4*, *Rph8, Rph9, Rph10, Rph12, Rph14,* and *Rph19*
5453 P−	Australia	560	*Rph1*, *Rph2, Rph4, Rph6, Rph9, Rph10,* and *Rph12*
5457 P−	Australia	626	*Rph1*, *Rph2, Rph3, Rph4, Rph6, Rph9, Rph10, Rph12,* and *Rph19*
5457 P+	Australia	612	*Rph1*, *Rph2, Rph3, Rph4, Rph6, Rph9, Rph10,* and *Rph12*
5672 P+	Australia	639	*Rph2*, *Rph4*, *Rph5*, *Rph6, Rph8, Rph9, Rph10*, *Rph12*, and *Rph19*
5477 P−	Australia	672	*Rph1, Rph*2, *Rph3, Rph4, Rph5, Rph6, Rph9, Rph10,* and *Rph12*
2654 P+	United States	17TX10B	*Rph1, Rph2, Rph4, Rph6, Rph8, Rph9, Rph11, Rph13,* and *Rph19*
3273 P−	United States	17WA26B	*Rph1, Rph2, Rph4, Rph5, Rph6, Rph8, Rph10, Rph11,* and *Rph14*

^#^Rust culture number stored at PBI Cobbitty.

** Tested for virulence on: *Rph1, Rph2, Rph3, Rph4, Rph5, Rph6, Rph7, Rph8, Rph9, Rph10, Rph11, Rph12, Rph13, Rph14, Rph15, Rph16, Rph19*, and *Rph25* (Australia; Park et al., 2015); *Rph1, Rph2, Rph3, Rph4, Rph5, Rph6, Rph7, Rph8, Rph9, Rph10, Rph11, Rph12, Rph13, Rph14, Rph15*, and *Rph19* (United States; Rouse et al., 2013).

Pathotype designations were based on virulence or avirulence responses of an isolate on the differential set using the octal notation system proposed by Gilmour (1973). Symbol P− or P+ was used to specify avirulence and virulence, respectively, on the barley cultivar Prior carrying *Rph19* (Park, 2003).

* The pathogenicity of these pathotypes for *Rph6* is unknown due to avirulence on *Rph2* in each and the presence of this gene in the *Rph6* differential tester Bolivia (*Rph2* + *Rph6*).

### Sowing and inoculations

#### Australia

The test lines and control genotypes (three lines/pot in clumps @ 8–10 seedlings/pot) and F_3_s (1 line/pot dispersed @ 20 seedlings/pot) were sown in 90 mm diameter pots containing Grange Horticultural^®^ soil premix comprised of 10% composted pine bark, 80% pine bark, 10% propagating sand, 1 kg/m^3^ gypsum, 1 kg/m^3^ superphosphate, 0.25 kg/m^3^ potassium nitrate, 0.25 kg/m^3^ nitroform, and 1.5 kg/m^3^ magrilime. Prior to sowing, all pots were fertilized with Aquasol @ 25 g/10 l of water. After sowing, pots were kept at 24°C in a temperature-controlled growth room. Ten-days old seedlings were inoculated with a *P. hordei* pathotype 5457 P+. A suspension mixture was prepared by adding 10 mg urediniospores /10 ml of isopar, a light mineral oil (Univar., Ingleburn, NSW, Australia), for 200 pots and the mixture was then atomized over the seedlings with a mist atomizer. Following inoculation, seedlings were incubated at 25°C in a dark chamber for 24 h. An ultrasonic humidifier was used to create mist in the incubation chamber. After 24 h of incubation, seedlings were shifted to microclimate rooms maintained at 24°C with natural light and an automated irrigation system.

#### United States

The three test lines were also rust tested in Minnesota, United States with two North American *P. hordei* pathotypes (17TX10B and 17WA26B). Test lines and control genotypes (four lines/pot in clumps @ 20 seedlings/pot) were sown in plastic pots (6.7-cm width × 6.7-cm length × 5.7-cm height) filled with vermiculite (Sun Gro Horticulture). Approximately 4 days after sowing and 1 week after inoculation, the pots were fertilized with a 20:20:20 NPK fertilizer @ 5 g/L of water. After sowing, pots were kept in a greenhouse maintained at 19°C–22°C with a photoperiod of 16 h facilitated by supplemental lighting. Approximately nine-days old seedlings were inoculated with a 15 mg urediniospores/0.75 ml Soltrol 70 lightweight mineral oil (ConocoPhillips Inc.) suspension inside a gelatin capsule from selected *P. hordei* isolates. Inoculations were facilitated by a custom rust inoculator (St. Paul machine shop, University of Minnesota) pressurized by an air pump (30 kPa). After inoculation, plants were placed under a fume hood to allow the oil to evaporate for 20 min. Plants were then placed in a dew chamber where humidity was maintained by ultrasonic humidifiers (V5100NS; Vicks) turned on for 2 min every 15 min for 16 h without light. Then, 400 W high-pressure sodium vapor lamps (LR217718; Kavita Canada Inc.) were turned on above the dew chambers that possessed a transparent plastic roof, allowing light penetration to the plants. After 2 h, the doors of the dew chambers were opened, and the plants were moved to a greenhouse maintained at 19°C–22°C with a photoperiod of 16 h facilitated by supplemental lighting.

### Disease scoring

Rust responses in Australia and United States were recorded using a “0”–“4” infection type scale (“0”-“hypersensitive flecks”; no sporulation and “1”–“4” increasing sporulation in the pustules) proposed by Park and Karakousis (2002). Plants were scored when the susceptible control Gus reached infection type (IT) of “3+” (typically 8–10 days after inoculation).

### DNA extraction, genotyping and targeted genotyping by sequencing

Genomic DNA was extracted from leaf tissues of single plants of F_3_ families for all three populations using a CTAB (Cetyl Trimethyl Ammonium Bromide) protocol (Fulton et al., 1995). Concentration of DNA was determined by using spectrophotometer (NanoDropTM, Biolab, Melbourne, VIC, Australia) and quality was determined by running all the samples on 0.8% agarose gel. To rule out the possible presence and involvement of *Rph7* and *Rph15*, all three AGG lines used as resistant parents in this study were genotyped with Indel markers closely linked to *Rph7* (Dracatos et al., unpublished) or a highly diagnostic KASP marker for *Rph15* (Chen et al., 2021). DNA from individual plants of F_3_ families from each of the three populations (AGG-396/Gus, AGG 397/Gus and AGG-403/Gus) showing monogenic inheritance with pathotype 5457 P+ was diluted to 100 ng/μl and sent to Agriculture Victoria, AgriBio Bundoora, Australia, for targeted genotyping by sequencing (tGBS) analysis on a fee-for service basis.

### Development of molecular markers (KASP and CAPS) for AGG-396/Gus

The target region identified through tGBS for the AGG-396/Gus F_3_ mapping population was enriched with both KASP and CAPS (Cleaved Amplified Polymorphic Sequences) markers. For KASP markers, SNPs identified in the target region were used directly to develop KASP assays by designing two allele-specific forward primers and one common reverse primer or vice versa using batch primer 3.^1^

KASP assays were performed in 96 well plates with an 8 μl reaction volume containing 4 μl of genomic DNA (10 ng/ul), 3.89 μl KASP mix (LGC Biosearch Technologies) and 0.11 μl of primer mix. All KASP reactions were conducted using a real time PCR machine-CFX96 (Biorad, United States) with 94°C for 15 min, 10 touchdown cycles at 94°C for 20 s, 65 to 58°C (reducing 0.6°C /cycle) and 35 cycles at 94°C for 20 s, 1 min at 55°C. Plates were read at 40°C and data were analyzed using allelic discrimination function.

For developing CAPS markers, sequence information for chromosome 2H based on Morex v2.0 (Mascher, 2019) was downloaded from IPK barley blast server and primers were designed to the pseudomolecules of chromosome 2H using primer 3 plus.^2^ PCR conditions for these markers were optimized and all markers were subsequently screened for polymorphism on both parental genotypes (AGG-396 and Gus). A 50 μl reaction was set comprising 15 μl of genomic DNA (10 ng/ μl), 10 μl Mi-Fi buffer, 0.5 μl of taq DNA polymerase (Bioline), 5 μl of each of forward and reverse primers (1.5 μM), and 14.5 μl of double distilled water. PCR conditions comprised an initial denaturation step at 95°C for 10 min, followed by 30 cycles with denaturation at 94°C for 30 s, annealing at 60°C for 30 s and extension at 72°C for 30 s. A final extension step of 10 min at 72°C was used.

The amplicons from parents (AGG-396 and Gus) were purified using Agencourt AMPure protocol- “000601v024” (Agencourt Bioscience Corporation). Forty microliters of AMPure XP was added to 40 μl of PCR product in a 96 well plate and mixed by pipetting. The samples were kept on a magnetic SPRI (solid-phase reversible immobilization) plate for 1 min. Liquid was eluted and DNA samples were washed twice with 120 μl of 70% ethanol to remove any contamination. Ethanol was discarded and samples were kept at room temperature for 5 min to evaporate any remaining liquid in the plate. One hundred microliters of 10 mM Tris (pH = 8) was added and mixed by pipetting. Samples were again kept on the SPRI magnetic plate for 1 min. Purified DNA (30 μl) was eluted and transferred to the new plate. In order to sequence, 8 μl purified PCR product was mixed with 4 μl of primer and sent to AGRF (Australian Genome Research Facility) for Sanger sequencing. For CAPS assays, PCR products were restricted using specific endonucleases as per the manufacturers protocol (New Biolab England, Australia).

**Figure 1:**
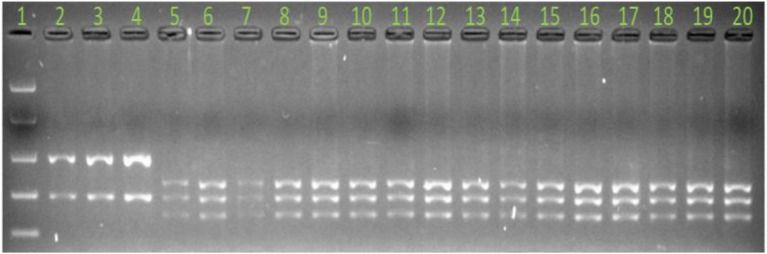
Marker validation on Australian barley cultivars with marker MLoc-70. Products were digested with restriction enzyme BSrI. Lane number 1 = easy ladder (Bioline). Lane numbers 2, 3, and 4 = PI 584760, Bowman + *Rph14* and resistant parent AGG-396, respectively. Lane numbers 5 to 7 = Gus, Bowman and Morex, respectively, and lane numbers 8 to 20 Australian Barley cultivars. Note the presence of susceptible allele for all cultivars shown in this image relative to lanes 2, 3, and 4, which are leaf rust resistant controls.

All CAPS primers were first used to amplify the parental genotypes (susceptible vs. resistant parent) and CAPS primers that were successfully amplified on the parents were converted to CAPS. Out of the 75 CAPS and 15 KASP markers designed (Supplementary File Tables S1, S2), 18 (3 KASP and 15 CAPS) were polymorphic between the parents and were used to genotype all F_3_ progeny (*n* = 105) for mapping of the *RphAGG396* resistance. Once flanking markers were identified, a further 600 F_2_ plants were genotyped with flanking markers for high resolution mapping of *RphAGG396*. Of the four markers that co-segregated with *RphAGG396* in the high-resolution mapping population, MLoc-70 was validated on 70 Australian barley cultivars (Figure 1; Supplementary File Table S3) that are known to lack *Rph14* (Singh et al., 2020).

### Gel electrophoresis

Digested PCR products were loaded onto 2% agarose gels. Each gel was prepared by mixing 5 g agarose in 250 ml of TAE buffer and boiling to dissolve the mixture. The gel was cooled under running tap water and then gel red was added (1 μl per 100 ml of solution). Five microliter loading buffer [98% formamide, 10 mM EDTA (pH 8.0), 0.05% Bromophenol blue and 0.05% xylene cyanol] was mixed to 15 μl digested PCR product and was spun down. 5 μl product was then loaded to each well using 2 kb Easy Ladder (Bioline) as a size reference. Electrophoresis was carried out at 110 volts for 60 min or more depending upon the size of digested products. Separated products were visualized using Gel-Doc IT imaging system (Model-M-26, Bioimaging Systems, CA, United States). The gel was scored as A = resistant, B = susceptible and H = heterozygous. Marker genotyping data was compared with the F_4_ phenotype.

### Sample size for initial mapping and high-resolution mapping

For initial mapping studies, 10 to 12 seeds of each F_3_ family were phenotyped. DNA was extracted from single leaf of each F_3_ family, and that DNA was used for marker genotyping. A single seed from each family was then advanced to the F_4_ generation and marker genotyping data was plotted against F_4_ phenotype. In case of *RphAGG396* mapping, all F_3_ families (*n* = 105) were used for phenotyping and genotyping. For fine mapping studies, 600 F_2_ plants were phenotyped and genotyped.

### Statistical analysis

F_3_ families were scored as non-segregating resistant (NSR—when all plants of individual family were resistant), non-segregating susceptible (NSS—when all plants of individual family were susceptible), or segregating (Seg—when both resistant and susceptible plants were found within a family). The data obtained were subjected to Pearson’s Chi-squared analysis (χ^2^) at significance level α = 0.05 to determine the goodness-of-fit of the observed ratios with expected genetic ratios. Online calculator Quickcalcs (GraphPad Software Inc., United States) was used to determine the *p*-values from χ^2^ values.

For tGBS mapping, the genotype calling trimmed sequence data was aligned against the IBSC genome assembly of Morex v1.0 using Nuclear software (GYDLE Inc.) to map each read to the best possible chromosome location. Associations were reported when at least 80% of the read length aligned, where three mismatches were tolerated in a window of 70 bases (i.e., ~96% identity). Genotypes were called when a SNP (Single Nucleotide Polymorphism) had a minimum read depth of 4, for SNPs that occurred in at least 4 samples, using custom perl scripts (a version 4.2 vcf file was generated). A marker was considered to be putatively linked with the target trait when one of the following criteria was met: minimum 50% call rate and minimum 70% allele fixation in the resistant progeny lines (i.e., fixed in resistant only); the susceptible progeny lines (i.e., fixed in susceptible only); minimum 50% call rate and minimum 70% allele fixation across each of the resistant and susceptible progeny lines (i.e., fixed in resistant and susceptible).

CAPS and KASP markers were designed within interval of 39 to 57 Mb (Mega bases) using Morex reference genome assembly v2.0. Sequence information of polymorphic KASP and CAPS primers is given in Tables 2, 3, respectively. The nucleotide sequences for both parents were analyzed for SNPs using Sequencher 5.1 software (Gene Codes, Ann Arbor, MI, United States). The identified SNPs were further subjected to dCAPS (Derived Cleaved Amplified Polymorphic Sequences) Finder 2.0 to identify restriction endonucleases.

**Table 2:** Sequence information for polymorphic KASP primers used to map *RphAGG396*.

Marker ID	Position^*^	SNP	Allele 1	Allele 2	Common primer
HvGBSv2-902	35.26	G/T	CCAGAAAAAGCCAGCTCAG	GCCAGAAAAAGCCAGCTCAT	CCAAAGGTGCATATCCGAAG
HvGBSv2-7015	45.83	T/G	CGAGAGCAAGAGAGGGGCT	AGAGCAAGAGAGGGGCG	TGTGGGCACGATGACTACAT
HvGBSv2-932	47.32	T/C	GCAACACAAGCTCGCCT	GCAACACAAGCTCGCCC	TGCATGCATTGGAAGAAGAG

* Positions (in million bases) are based on Morex reference genome assembly v.2.

Sequence information of primers provided here are without HEX and FAM.

**Table 3:** Sequence information for polymorphic CAPS primers and respective restriction enzymes used to map *RphAGG396*.

Marker ID	Locus	Position^*^	Forward primer sequence	Reverse primer sequence	Endonucleases
MLoc-6	r2.2HG0091750	36.48	TCCTCTCTGAGATGGCAACA	GATCGACGGACCTTGAAGAC	RsaI
MLoc-10	r2.2HG0092260	38.50	CGAGGAGCTCTCCACCTACA	GCTGGAGAGCAAACAGGAAC	MboI
MLoc-13	r2.2HG0092810	40.50	CCTTGTCCGTGATGCAACTA	CCCCTATCGGAGGAGGTATT	BcII
MLoc-28	r2.2HG0092860	40.75	ATTGGTTGCGCTTTGCTATC	ATCATAGGTTTCGCCACGTC	MaeIII
MLoc-29	r2.2HG0093020	41.43	GCAAAGACTCCCCTTTAGGC	CCGCTGCTAGAACTTTCAGG	BsrDI
MLoc-31	r2.2HG0093020	41.43	AAGTTGAAGGTCCGTGGATG	CTCTAGAGAAGGCGGGAGGT	DdeI
MLoc-36	r2.2HG0093070	41.80	TGGTTAGCTACGAGGGGAGA	ATGACACATGCAAACCCGTA	PstI
MLoc-38	r2.2HG0093100	41.81	GCATCGGCTCTACCTCGTC	CGATGGTAGCCCATTCAATC	MboII
MLoc-39	r2.2HG0093120	41.83	CTCAATTTCTTCCGGACCAG	TTGCCGGCAGTTTACCTAAC	MboII
MLoc-41	r2.2HG0093160	41.92	GGACCATTTCTTTGCTGGAA	AGCAAAACTGCAGAGGGAAA	BsmAI
MLoc-42	r2.2HG0093180	41.98	AAGCTAAGCAGCTCGAAACG	CAGAATAGCGCACTTGTTGG	BsmAI
MLoc-44	r2.2HG0093260	42.10	GTGTCCTCCGTCGTCACC	GGCTTTGGCTGCTTGACTAT	Alu1
Mloc-70	r2.2HG0093030	41.43	GGGTCTCATCGAGAACCTCA	CACGGTCTTCCCATTACCAT	Bsr1
Mloc-71	r2.2HG0093030	41.43	GTAATGGGAAGACCGTGCAG	CCCTGTACCTCCAATGCCTA	Aci1
Mloc-74	r2.2HG0093120	41.83	CGTTAGGACGTGCGTTCTGT	CTGGTCCGGAAGAAATTGAG	Apo1

* Positions (in million bases) are based on Morex reference genome assembly v2.0.

## Results

### Infection type response and genotyping of the parents

The three resistant parents investigated in this study produced very low to intermediate ITs, while the susceptible parent Gus produced a high IT with eight Australian pathotypes (Table 4). The parental line AGG-397 showed relatively lower infection type compared to AGG-396 and AGG-403 with pathotypes 200 P− and 220 P+ suggesting the presence of an additional gene in AGG-397. All three resistant parents carried susceptibility alleles when genotyped with markers linked to *Rph7* (Dracatos et al., unpublished) and *Rph15* (Chen et al., 2021), supporting the likely absence of *Rph7* and *Rph15* (effective against all known Australian pathotypes) in the resistant parents (Supplementary File Table S4).

**Table 4:** Infection types produced by AGG-396, AGG-397, and AGG-403 when tested against eight *Puccinia hordei* pathotypes in the greenhouse.

	Pathotype							
Line	200 P−	220 P+	253 P−	5652 P+	5610 P+	5453 P−	5457 P−	5457 P+
AGG-396	;1 + C	;12C	;12 + CN	;1 + CN	;1CN	;1 + CN	;1 N	;1 + N
AGG-397	;N	;N	;1 + N	;1CN	;1CN	;1-N	;1-CN	;1CN
AGG-403	;1 + C	;12C	;12C	;1 + CN	;1CN	;1 + CN	;1 + CN	;1 + CN
Gus (Sus)	3+	3+	3+	3+	3+	3+	3+	3+

Infection types are based on a “0”–“4” scale (Park and Karakousis, 2002). Symbol “;” represents hypersensitive flecks, “1” represents small uredinia enclosed by necrotic tissues, “2” represents medium size uredinia enclosed by chlorotic and/or necrotic tissues, “3” represents large size uredinia with or without chlorosis and “3+” represents susceptible host, “N” represents necrosis, “C” represents chlorosis. Symbols “+” and “−” represent higher and lower infection types than normal, respectively. Gus was used as a susceptible check.

### Phenotyping and genetic analysis of mapping populations

The F_1_s derived from three crosses showed infection type similar to that of the respective resistant parents involved indicating that the resistance in all three lines is dominant. The F_3_ families derived from all three populations segregated for resistant (;CN to;12CN) and susceptible (3+) IT responses when tested with pathotype 5457 P+. Chi squared analysis of phenotypic data of F_3_ families revealed a goodness of fit of the data for a single gene segregation conforming to a 1:2:1 (NSR:Seg:NSS) genetic ratio (*p* > 0.6 to 0.8) in all three populations (Table 5). The pooled analysis based on resistant (R) and susceptible (S) individuals within segregating lines showed goodness of fit for a 3R:1S ratio in all three populations (AGG-396/Gus = 603R:227S, *p* > 0.11; AGG-397/Gus = 578R: 211S, *p* > 0.25; AGG403/Gus = 680R:233S, *p* > 0.71) confirming that resistance in all three lines is dominant. To determine if the difference in IT response of AGG-397 relative to AGG-396 and AGG-403 is due to presence of an additional gene, the F_3_s of AGG-397/Gus were also tested with pathotype 200 P−. The population segregated (27NSR:52Seg:23NSS) in a similar fashion with this pathotype as that with pathotype 5457 P+ and conformed to a genetic ratio (1:2:1, *p* > 0.8) expected for a single dominant gene.

**Table 5:** Distribution and Chi-squared analysis of F_3_ families derived from crosses between AGG-396/Gus, AGG-397/Gus, and AGG-403/Gus when tested against *Puccinia hordei* pathotype 5457 P+ in greenhouse.

Population	NSR	Seg	NSS	Tested ratios	χ^2^	Value of *p*
AGG-396/Gus	24	57	23	1:2:1	0.98	0.61
AGG-397/Gus	27	52	23	1:2:1	0.35	0.83
AGG-403/Gus	27	66	33	1:2:1	0.85	0.65

NSR, non-segregating resistant; Seg, segregating; NSS, non-segregating susceptible.

### Locating genomic regions associated with resistance to *Puccinia hordei*

**Figure 2:**
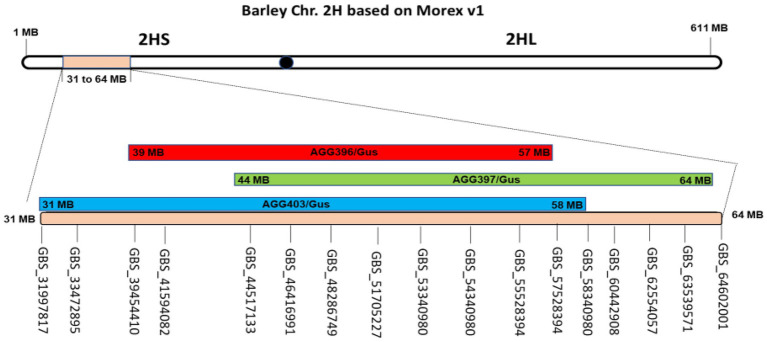
Genomic regions for AGG-396 (red), 397 (green), and 403/Gus (blue) F_3_ populations identified on the short arm of chromosome 2H through tGBS. For AGG-396/Gus, AGG-397/Gus and AGG-403/Gus, the intervals linked to resistance were found from 39 to 57, 44 to 64, and 31 to 58 Mb on 2HS, respectively.

To determine chromosomal regions associated with the observed leaf rust resistance, all three populations (AGG-396/Gus, AGG-397/Gus, and AGG-403/Gus) were genotyped using tGBS. In all three populations, markers linked to resistance were detected on the short arm of chromosome 2H between 39 and 57 Mb, 44 and 64 Mb, and 31 and 58 Mb, respectively (Figure 2) based on the Morex reference assembly v.1 (Mascher et al., 2017).

### Relationship of *RphAGG396*, *RphAGG397*, and *RphAGG403* with previously reported genes on 2HS

The leaf rust resistance carried by three lines (tentatively designated *RphAGG396*, *RphAGG397*, and *RphAGG403*) was detected in the same region as that reported previously for genes *Rph14*, *Rph15*, and *Rph16* on chromosome 2HS (Martin et al., 2020; Chen et al., 2021). The possibility of the resistance in these lines being *Rph15*/*Rph16* (demonstrated as the same genes, Chen et al., 2021) was rejected based on all three lines being negative for a diagnostic marker for *Rph1*5. The recently reported physical location of *Rph14* (45.7–57.3 Mb) reported by Martin et al. (2020) using GBS SNPs suggested the possible presence of *Rph14* in these lines.

**Figure 3:**
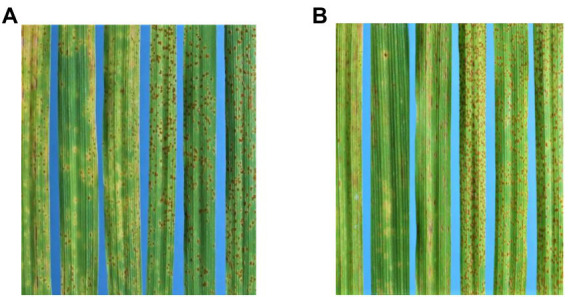
**(A)** From left to right, infection types of AGG-396, 397, 403, PI584760, Bowman + *Rph14*, and Bowman, respectively, with 5610 P+. **(B)** Infection types of AGG-396, 397, 403, PI584760, Bowman + *Rph14*, and Bowman, respectively, with 210 P+.

To assess this possibility more critically, the parents AGG-396, 397, and 403 along with the *Rph14* donor line-PI 584760, a line near isogenic to Bowman carrying *Rph14*, Bowman and Gus were phenotyped with additional *P. hordei* pathotypes 5610 P+ (Figure 3A) and 210 P+ (Figure 3B), both considered to carry virulence for *Rph14* (Park RF, unpublished). AGG-396, 397 and 403 were all resistant (IT ranging from “;+N” to “;1+CN”) to pathotypes 5610 P+ and 210 P+, while Bowman, Bowman + *Rph14* and PI 584760 were susceptible (IT “3” to “3+”) to both pathotypes indicating that the locus involved in three test lines is distinct from *Rph14*. To further validate these results, the three lines were sent to Minnesota, United States and tested with two additional isolates 17TX10B (avirulent on *Rph14*) and 17WA26B (virulent on *Rph14*). The tests however showed specificity allied to *Rph14*; all three lines being susceptible with isolate 17WA26B and resistant with isolate 17TX10B. The incompatible IT response of AGG-396, AGG-397 and AGG-403 to Australian *Rph14*-virulent pathotypes 5610 P+ and 210 P+ in contrast to *Rph14*-specific response with the North American pathotypes implied that resistance in these lines is likely conditioned either by a distinct allele of *Rph14* or a gene very closely linked to *Rph14* which is not effective to North American isolate 17WA26B.

### Mapping *RphAGG396*

Detection of the same physical region linked to resistance in AGG-396, AGG-397, and AGG-403 (based on the Morex reference genome assembly) suggested the possible involvement of the same locus in the three lines. Based on this assumption, further experiments were conducted to fine map only the resistance locus *RphAGG396*. This locus was preferred because it was the smallest physical region (18 Mb) compared to *RphAGG397* (20 Mb) and *RphAGG403* (22 Mb).

**Figure 4:**
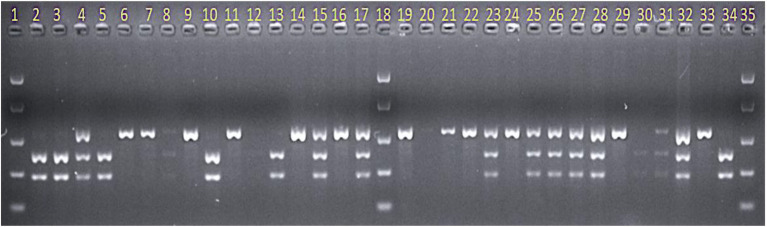
Gel image showing screening of the AGG-396/Gus derived F_3_ population with CAPS marker Mloc-39 when digested with restriction enzyme MboII. Lane numbers 1, 18, and 35 = Easy Ladder (Bioline). Lane numbers 33 and 34 = resistant parent AGG-396 and susceptible parent Gus, respectively. All other lanes show F_3_ progeny from the AGG-396/Gus population.

**Figure 5:**
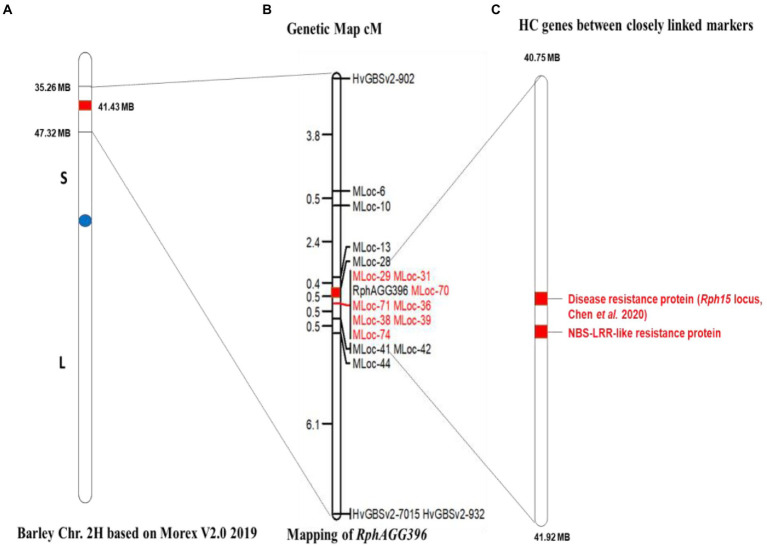
Genetic and physical map for *RphAGG396* based on the Morex genome assembly 2019 (v2). **(A)** Barley chromosome 2H showing the physical window between 35 and 47 Mb for *RphAGG396* based on polymorphic KASP and CAPS markers, **(B)** fine map of *RphAGG396* placing the locus between MLoc-28 and MLoc-41 within a physical interval of 1.17 Mb. Markers co-segregating with *RphAGG396* are highlighted red **(C)** predicted NLRs genes (red highlighted) between MLoc-28 and MLoc-41.

Two KASP markers, HvGBSv2-902 and HvGBSv2-932, defined a genetic distance of 3.8 cM (with 8 recombinants) proximal, and 7.1 cM (with 15 recombinants) distal to *RphAGG396*, respectively. The *RphAGG396* locus was further saturated with 15 CAPS markers developed between the two flanking markers (Figure 4). Integration of marker data to F_4_ phenotypes resulted in the identification of two critical recombinants, placing *RphAGG396* between two closely linked markers Mloc-28 (40.75 Mb) and Mloc-41 (41.92 Mb) at a genetic distance of 0.47 cM at proximal and distal ends (Figure 5). The physical window between the newly determined flanking markers was narrowed to a 1.17 Mb region based on the Morex v2.0 reference genome assembly (Mascher, 2019).

### High resolution mapping of *RphAGG396* and marker validation

In order to enhance map resolution, 600 F_2_ plants were genotyped with the markers Mloc-28 and Mloc-41, and nine recombinants were identified. Phenotyping and genotyping of these recombinants placed the gene at genetic distances of 0.5 and 0.2 cM between Mloc-28 and Mloc-41, respectively. Eight markers (MLoc-29, MLoc-31, MLoc-36, MLoc-38, MLoc-39, MLoc-70, MLoc-71, and MLoc-74) were found to co-segregate with *RphAGG396* in a low resolution F_3_ mapping population (Figure 1), among which four (MLoc-29, MLoc-31, MLoc-70, and MLoc-71) co-segregated with the resistance phenotype in a high resolution F_2_ population (AGG396/Gus).

Marker MLoc-70, derived directly from the sequence of putative candidate gene-r2.2HG0093030 in the target interval was validated on 70 Australian barley cultivars (Supplementary File Table S3) that are considered to lack *Rph14* (Singh et al., 2020). MLoc-70 amplified susceptible alleles in all 70 Australian barley cultivars and two leaf rust susceptible controls (Gus and Bowman) and the resistant allele in PI 584760, Bowman + *Rph14* and AGG-396. Furthermore, the susceptible allele was amplified from Morex DNA suggesting that the *RphAGG396* candidate gene may not be present in the reference genome (Figure 1).

### Gene annotation

The genomic region between Mloc-28 and Mloc-41 was searched for high confidence (HC) genes using the Morex reference genome assembly v.2. The region between these markers carries 17 HC genes (Table 6). The functional annotation of these HC genes was retrieved using IPK Gatersleben.^3^ The annotated genes in the target interval include two NLR genes, r2.2HG0093020, which is a locus for *Rph1*5 as reported by Chen et al. (2021), and r2.2HG0093030, which encodes an NBS-LRR-like resistance protein.

**Table 6:** High confidence genes annotated in the target interval between the flanking markers Mloc-28 and Mloc-41.

Gene ID	Start position (bps)	End position (bps)	Functional annotation
r2.2HG0092910	41,105,597	41,105,929	Zinc finger MYM-type protein
r2.2HG0092920	41,109,556	41,112,246	Hfr-2-like protein
r2.2HG0092930	41,282,170	41,284,359	Pentatricopeptide repeat-containing protein
r2.2HG0092940	41,285,574	41,287,096	F-box family protein
r2.2HG0092970	41,297,210	41,298,340	60S ribosomal protein L22
r2.2HG0092980	41,360,395	41,363,393	UPF0235 protein
r2.2HG0092990	41,365,081	41,367,971	DNA-directed RNA polymerases I, II, and III subunit rpabc3
r2.2HG0093000	41,368,892	41,372,924	UDP-glucuronate decarboxylase
r2.2HG0093020^*^	41,433,854	41,437,075	Disease resistance protein (TIR-NBS-LRR class)
r2.2HG0093030^*^	41,437,202	41,439,356	NBS-LRR-like resistance protein
r2.2HG0093040	41,530,676	41,531,044	B0809H07.3 protein
r2.2HG0093050	41,675,798	41,676,166	B0809H07.3 Protein
r2.2HG0093060	41,735,236	41,737,909	Hypoxia-responsive family protein-like
r2.2HG0093070	41,809,611	41,809,988	UDP-4-amino-4-deoxy-L-arabinose--oxoglutarate Aminotransferase
r2.2HG0093100	41,815,749	41,816,882	Kelch repeat-containing protein
r2.2HG0093120	41,833,899	41,835,608	F-box family protein
r2.2HG0093150	41,913,620	41,917,079	Splicing factor-like protein

* NLR genes based on Morex reference genome assembly v.2.

## Discussion

This study was conducted to characterize and map leaf rust resistance in three Israeli barley lines (AGG-396, AGG-397, and AGG-403) based on previous results that suggested the lines carried uncharacterised all stage resistance (Mehnaz et al., 2021b). All three lines originated from the same geographical area and produced very similar infection types with all test pathotypes, and it was therefore hypothesized that the resistance in all three lines is likely conferred by the same resistance locus. Targeted genotype by sequencing (tGBS) conducted in this study supported our hypothesis as resistance was mapped to the same overlapping genomic region on the short arm of chromosome 2H in three mapping populations (AGG-396/Gus: 39–57 Mb; AGG-397/Gus: 44–64 Mb; AGG-403/Gus: 31–58 Mb).

Previously reported *Rph* genes on the short arm of chromosome 2H include *Rph14* (Golegaonkar et al., 2009), *Rph15* (Weerasena et al., 2004; Chen et al., 2021), *Rph16* (Ivandic et al., 1998), and *Rph17* (Pickering et al., 1998). Several previous studies conducted to determine the genetic relationship between the *Rph* genes on chromosome 2HS established that *Rph14* and *Rph16* are either allelic or closely linked to *Rph15* (Ivandic et al., 1998; Weerasena et al., 2004) and *Rph14* is independent of *Rph15* (Chicaiza, 1996) or closely linked (Derevnina et al., 2015). All these studies were conducted prior to the release of the Morex reference genome assembly of barley and therefore the exact physical location of the genes was not known. However, a recent study by Martin et al. (2020) placed *Rph14* and *Rph15* on 2HS from 45.7 to 57.3 Mb and 44.8 to 57.3 Mb in the Morex reference genome v.1 based on GBS-based SNP markers. Their findings also established that *Rph14* and *Rph15* are closely linked, independent genes. The latest findings in this context using Illumina whole genome sequencing in donor lines for *Rph14*, *Rph15*, and *Rph16* concluded that *Rph14* is independent from *Rph15* and that *Rph15* and *Rph16* are the same gene (Chen et al., 2021).

The three resistant lines investigated in this study tested negative for markers linked to *Rph15* (Chen et al., 2021), therefore *Rph15*/*16* is most probably not present in these lines. Based on different pathotypic specificity (observed with Australian pathotypes 5610 P+ and 210 P+) compared to the *Rph14* donor accession (PI 584760), and on other hand *Rph14*-specificity with North American pathotypes, it can be concluded that the resistance locus in AGG-396, 397, and 403 could be an allele of *Rph14* or an independent locus which is not effective against the *Rph14*-virulent North American pathotype used. However, further confirmation is recommended through a test of allelism between the three AGG lines and *Rph14* donor stock. Sequencing of AGG lines and PI 584760 (*Rph14*) may also help in revealing any differences at nucleotide level and understanding the nature of the underlying resistance.

In this study, we successfully fine mapped *RphAGG396* and narrowed down the physical region to 1.17 Mb. Functional annotation of the genes in the identified target region detected 17 high confidence genes including two NLR genes (r2.2HG0093020 and r2.2HG0093030). Among various known classes for resistance genes in plants, the most prevalent and commonly identified class of genes providing resistance to several pathogens encode immune receptor proteins that contain a nucleotide-binding site (NBS) and a leucine-reach repeat domain (referred to as NLRs; Dangl and Jones, 2001; Tan and Wu, 2012). Most disease resistance genes in cereals isolated so far belong to this class of gene, for example, *Rph1* (Dracatos et al., 2019) and *Rph15* (Chen et al., 2021) in barley and leaf rust resistance genes *Lr1*, *Lr10*, and *Lr21* (Feuillet et al., 2003; Huang et al., 2003; Cloutier et al., 2007), stem rust resistance genes *Sr33, Sr35*, and *Sr50* (Periyannan et al., 2013; Saintenac et al., 2013; Mago et al., 2015) and yellow rust resistance genes *Yr5*, *Yr10*, and *Yr27* (Liu et al., 2014; Marchal et al., 2018; Athiyannan et al., 2022) in wheat.

The two annotated NLR genes (r2.2HG0093020 and r2.2HG0093030) were identified as the best candidates for *RphAGG396,* the former was however reported as *Rph15* (Chen et al., 2021). Martin et al. (2020) reported that *Rph14* and *Rph15* are closely linked genes between 45 and 57 Mb in reference genome assembly v.1. The two markers (MLoc-29 and MLoc-31) designed to the CDS (coding sequences) of NLR gene-r2.2HG0093020 and further two (MLoc-70 and MLoc-71) designed to the CDS of second NLR gene-r2.2HG0093030 co-segregated with *RphAGG396* in a high-resolution mapping population. Therefore, these two NLRs could be candidate genes for *RphAGG396.* As previous studies also reported that both *Rph14* and *Rph15* are tightly linked genes (Derevnina et al., 2015; Martin et al., 2020; Chen et al., 2021) and the CDS of two identified NLR genes in this study are at 5 kb distance from one another in Morex reference genome v.2, it was not surprising that all four markers co-segregated with both of these NLR genes. It is probable that there are cultivar specific genes possibly not present in Morex that may represent both *Rph14* and *RphAGG396*. NLRs clusters in genomes evolve mainly through divergent evolution and lineage specific duplication events (Michelmore and Meyers, 1998; Zhong et al., 2018).

Of four co-segregating markers, MLoc-70 was validated on 70 Australian barley cultivars (Supplementary File Table S3) that are believed to lack *Rph14* (Singh et al., 2020). The susceptible allele was amplified in all 70 barley cultivars and the leaf rust susceptible controls Gus and Bowman, while the resistance allele was amplified in PI 584760, Bowman + *Rph14* and the resistant parent AGG-396, suggesting that this marker is highly predictive for *RphAGG396* and can be used for marker-assisted selection.

## Data availability statement

Existing datasets are available in a NCBI GenBank publicly accessible repository (https://www.ncbi.nlm.nih.gov). Publicly available datasets were analyzed in this study. This data can be found at: https://www.ncbi.nlm.nih.gov - accession numbers OP584888, OP584889, OP584890, OP584891, OP584892, OP584893, OP584894, OP584895, OP584897, OP584898, OP584899, OP584900, OP584901, and OP584902.

## Author contributions

MM lead the studies. DS designed the studies. DS, PMD, and RP supervised the studies. RP provided all Australian pathotypes. MM and DS performed phenotyping and data analysis and wrote the manuscript. MR conducted phenotyping in United States. MM, PMD, KF, and HXD performed recombination and/or tGBS analysis. All authors contributed to the article and approved the submitted version.

## Conflict of interest

The authors declare that the research was conducted in the absence of any commercial or financial relationships that could be construed as a potential conflict of interest.

## Publisher’s note

All claims expressed in this article are solely those of the authors and do not necessarily represent those of their affiliated organizations, or those of the publisher, the editors and the reviewers. Any product that may be evaluated in this article, or claim that may be made by its manufacturer, is not guaranteed or endorsed by the publisher.

## Footnotes

^1^ https://probes.pw.usda.gov/cgi-bin/batchprimer3/batchprimer3.cgi

^2^ http://www.bioinformatics.nl/cgi-bin/primer3plus/primer3plus.cgi

^3^ webblast.ipk-gatersleben.de/barley_ibsc/
