# Supplementary material for: A novel locus conferring resistance to Puccinia hordei maps to the genomic region corresponding to Rph14 on barley chromosome 2HS
Source: Front Plant Sci. 2022 Oct 6;13:980870. doi: 10.3389/fpls.2022.980870 (PMC9583899; doi:10.3389/fpls.2022.980870)
Supplement: Supplementary file 1 [file Table_1.docx]

**Supplementary File Table S1:** Details of 75 CAPS markers designed to fine map *RphAGG396*.

| **Marker ID** | **Locus** | **Position*** | **Forward primer sequence** | **Reverse primer sequence** |
| --- | --- | --- | --- | --- |
| MLoc-1 | r2.2HG0091040 | 31.92 | CTAGCTCCAGGTCCCATGAC | AGGGCAACAGGAGAGGTAGG |
| MLoc-2 | r2.2HG0091320 | 32.84 | GAGGAAAAGTCATGCACACC | CACCATTAGGCCAAGGAGAA |
| MLoc-3 | r2.2HG0091410 | 34.06 | CTCCTCCTCCGGTTCGAT | AAGTCGTCGAGAAAGGCGTA |
| MLoc-4 | r2.2HG0091490 | 34.89 | CACCTGCAGGACCAGCTC | CCGGGAAGGTGAGGTAAATC |
| MLoc-5 | r2.2HG0091660 | 35.95 | GGAGGCCTTGTCTGGTGAG | GCGACAACGCCGTATACTTT |
| MLoc-6 | r2.2HG0091750 | 36.48 | TCCTCTCTGAGATGGCAACA | GATCGACGGACCTTGAAGAC |
| MLoc-7 | r2.2HG0091910 | 37.29 | CAAACACCTCCCGCTGTTAT | CCATCTCAAGACAGCAATGG |
| MLoc-8 | r2.2HG0091910 | 37.29 | TATGGATCACCAGCTGCATT | TAAAATGGAAGCAGCCCAAT |
| MLoc-9 | r2.2HG0092020 | 37.87 | TCCAAGACGTTTGTTGCTTG | AGGGATGACAAGGGGACAG |
| MLoc-10 | r2.2HG0092260 | 38.5 | CGAGGAGCTCTCCACCTACA | GCTGGAGAGCAAACAGGAAC |
| MLoc-11 | r2.2HG0092390 | 39.02 | GTGAACGGCGAGGACCTG | CGGGCTGCTTCACATCAG |
| MLoc-12 | r2.2HG0092610 | 39.8 | GCCAACCTCCGTAGATCACT | CTCTTGTCGGAGGACACGTT |
| MLoc-13 | r2.2HG0092810 | 40.5 | CCTTGTCCGTGATGCAACTA | CCCCTATCGGAGGAGGTATT |
| MLoc-14 | r2.2HG0092920 | 41.1 | AGAAGCAGCTGCCTGAACAT | ACAGAAACCGTCTGATGCAA |
| MLoc-15 | r2.2HG0092930 | 41.28 | AACTCTGGACCAGCATGGAG | GCCTAGGCATCAAAGTGTGC |
| MLoc-16 | r2.2HG0093300 | 42.26 | ACGTCATGTGGTTCAGCAAC | CTGTGATCTGCGGACTTCAA |
| MLoc-17 | r2.2HG0093450 | 43.02 | GATATTCAAGCTGGGGCAGA | TTTGTTGTTTCCATCGTTGA |
| MLoc-18 | r2.2HG0093450 | 43.02 | GTTGTGGACGCATTTGATCC | ACGGTAGCTGCTGTTGGTCT |
| MLoc-19 | r2.2HG0093610 | 43.73 | TAGGCAGGCTATGGCCTAAA | ATGTGGTTCCTGTCCTCCAG |
| MLoc-20 | r2.2HG0093890 | 44.53 | TGGAGTTGGATTGGGAGAGT | ACCAAAGCACTTCCATCACC |
| MLoc-21 | r2.2HG0093960 | 45.19 | GCTTGGAGTTGAAAGGTGGT | CCTCTCTTCCACCCCAGAAT |
| MLoc-22 | r2.2HG0094010 | 45.49 | CGAAGGGTCTGGAGTACCTG | ACCATCATTTTCGCCGTAAG |
| MLoc-23 | r2.2HG0094120 | 46.14 | AGGGTTAGAGGGCAGGACAC | CTCCATCACCAGGTCGTACA |
| MLoc-24 | r2.2HG0094190 | 46.35 | GTCTTCGCCTCATCTCTTGC | GTTGAGAGAGACCCCCAACA |
| MLoc-25 | r2.2HG0094420 | 47.26 | CCCGCTGAAGAATATTGGAA | AAGCACGAGGTTCCGTTTTA |
| MLoc-26 | r2.2HG0094480 | 47.69 | TCCAGCTCTTCGACCTCAAC | TTGGCCTCCAAAATTCAAAC |
| MLoc-27 | r2.2HG0092840 | 40.6 | CCCGACAAATGGCATCTAAG | CGGGTAAGAGGTGGAAGATG |
| MLoc-28 | r2.2HG0092860 | 40.75 | ATTGGTTGCGCTTTGCTATC | ATCATAGGTTTCGCCACGTC |
| MLoc-29 | r2.2HG0093020 | 41.43 | GCAAAGACTCCCCTTTAGGC | CCGCTGCTAGAACTTTCAGG |
| MLoc-30 | r2.2HG0093020 | 41.43 | CAGAAATGGAGGAGCACTCG | TAGCCACTCCCACGCTATTC |
| MLoc-31 | r2.2HG0093020 | 41.43 | AAGTTGAAGGTCCGTGGATG | CTCTAGAGAAGGCGGGAGGT |
| MLoc-32 | r2.2HG0092930 | 41.28 | TAGCATCACTGCCGACTCTG | AGCAAGCCAAAAACCCTTCT |
| MLoc-33 | r2.2HG0092970 | 41.29 | CGTCGCTGGAGAAGTTCCT | TACCGGAGCTCGTACACGTT |
| MLoc-34 | r2.2HG0092980 | 41.36 | CCCCCTTGCATTACACAGTT | ATCCCCTTTGAAGGGTCATC |
| MLoc-35 | r2.2HG0092990 | 41.36 | TTTTTCCTCTCCTGCCAGTC | GGAGTCCCATCCAGATTCAA |
| MLoc-36 | r2.2HG0093070 | 41.8 | TGGTTAGCTACGAGGGGAGA | ATGACACATGCAAACCCGTA |
| MLoc-37 | r2.2HG0093100 | 41.81 | GCACCAGATCCTGGTCCAT | ATCCAACGAAATGCAGTGTG |
| MLoc-38 | r2.2HG0093100 | 41.81 | GCATCGGCTCTACCTCGTC | CGATGGTAGCCCATTCAATC |
| MLoc-39 | r2.2HG0093120 | 41.83 | CTCAATTTCTTCCGGACCAG | TTGCCGGCAGTTTACCTAAC |
| MLoc-40 | r2.2HG0093150 | 41.91 | TTCTTCTGCATCATGGCAAG | CTACGGCTGCGAGAATAGGA |
| MLoc-41 | r2.2HG0093160 | 41.92 | GGACCATTTCTTTGCTGGAA | AGCAAAACTGCAGAGGGAAA |
| MLoc-42 | r2.2HG0093180 | 41.98 | AAGCTAAGCAGCTCGAAACG | CAGAATAGCGCACTTGTTGG |
| MLoc-43 | r2.2HG0093180 | 41.98 | TGTCAGATGGGAAGCACCAT | CAATTTCTTTGCCAGCATCC |
| MLoc-44 | r2.2HG0093260 | 42.1 | GTGTCCTCCGTCGTCACC | GGCTTTGGCTGCTTGACTAT |
| MLoc-45 | r2.2HG0093280 | 42.2 | TTCGTCCTCTGGACTGGACT | GGACTTGGGCTGCTTGACTA |
| MLoc-46 | r2.2HG0093290 | 42.21 | GAGGAAGCGCAAGTTCCTG | GCGCTTTTGGTTCTGTCTGT |
| MLoc-47 | r2.2HG0093310 | 42.26 | GGAGAGATAGCACCGACGAA | GGCTCATGCCGCACTATTAT |
| MLoc-48 | r2.2HG0093320 | 42.44 | TGGTGAAGCTTATCCAAAAGG | TGAATTCCAATGTCCAAACG |
| MLoc-49 | r2.2HG0093330 | 42.45 | TGAAGATGCACGACGAGTCT | TCAGGGTGAGTCACATGAGC |
| MLoc-50 | r2.2HG0093350 | 42.49 | TCAGAAGCTCCTCCCAGCTA | CATGTACTTGCGCTCGATGT |
| MLoc-51 | r2.2HG0093390 | 42.63 | GTACACTCTTCGCCGTCGTT | CGATTCATGAGCCCGTAGAC |
| MLoc-52 | r2.2HG0093400 | 42.64 | TTGCCACGGAGAATGAATTT | TACCAGGTGGACCAAAAAGC |
| MLoc-53 | r2.2HG0093400 | 42.64 | AGCAGTGATGTCCGTCCTCT | TTCACCGTAAAGCTCGTTCC |
| MLoc-54 | r2.2HG0093420 | 42.83 | GCCGGAGATTACTCCTCCTC | AGACACCGCCAACTCCTAGA |
| MLoc-55 | r2.2HG0093440 | 42.99 | GGAGCTGGCCAGGGAGAT | CTTGGCCCCAAGATCCATAG |
| MLoc-56 | r2.2HG0093490 | 43.18 | AGCATCCTCCACGATCAAAA | GACCCGCACTTGAACTTCTC |
| MLoc-57 | r2.2HG0093590 | 43.49 | ACACGATCCTTGTCGATGGT | ATTGACACAGTGCCCATGAA |
| MLoc-58 | r2.2HG0093600 | 43.62 | AGCAAAGCTCCACTCTCGAA | GACTTGAGCAAACGCCTGAC |
| MLoc-59 | r2.2HG0093640 | 43.74 | ATAACTGCCAAGGACCATGC | TGCTTGATCTCAGCCCTCTT |
| Mloc-60 | r2.2HG0092820 | 40.51 | TTGCTGGATACCTGCCTTTT | AAGTTACCCACGACGCCTAC |
| Mloc-61 | r2.2HG0092860 | 40.76 | CTGGCGAAGTACTGGCTCTC | CGGAGGTTCTGGATGCAAT |
| Mloc-62 | r2.2HG0092870 | 40.8 | AGCAGATGCTATCCCTGCAC | CATGAGGGGGTTGTTGAGAT |
| Mloc-63 | r2.2HG0092920 | 41.1 | ATGCACCCTGTTTAAGCACA | TGCAAGTAAGGACGTTTGGA |
| Mloc-64 | r2.2HG0092940 | 41.28 | GGGAGATCATCCTTTGCGTA | GTCAATCATCAGGAGCACGA |
| Mloc-65 | r2.2HG0092940 | 41.28 | TATCGGTTATCGTCGGCTTC | GCGAGCAAGAGGCACTAGAT |
| Mloc-66 | r2.2HG0092980 | 41.36 | GACGACCCACACGTACTCG | CCTCGCAAGATGCTTTGTCT |
| Mloc-67 | r2.2HG0092980 | 41.36 | GCAGAAAATGCCAAAGAGGA | CCACAGAACTCAGTGCTCCA |
| Mloc-68 | r2.2HG0092990 | 41.36 | AGGACATCTTCCACGTGACC | GGAGCTAAAACCATGGTGAA |
| Mloc-69 | r2.2HG0093000 | 41.37 | ATAGCGTGATCGTGGTGGAC | CCCGTAGGTGTTGAAGATCC |
| Mloc-70 | r2.2HG0093030 | 41.43 | GGGTCTCATCGAGAACCTCA | CACGGTCTTCCCATTACCAT |
| Mloc-71 | r2.2HG0093030 | 41.43 | GTAATGGGAAGACCGTGCAG | CCCTGTACCTCCAATGCCTA |
| Mloc-72 | r2.2HG0093030 | 41.43 | GGATGACAGCTGGCAAATCT | CATGATGCGTCCACTACGTC |
| Mloc-73 | r2.2HG0093110 | 41.82 | ATTGATCAGGTCCGCTTGAC | GGAAATCGCAAGCTGGAAC |
| Mloc-74 | r2.2HG0093120 | 41.83 | CGTTAGGACGTGCGTTCTGT | CTGGTCCGGAAGAAATTGAG |
| Mloc-75 | r2.2HG0093150 | 41.91 | GTTTGAAGATCCCCGTGATG | CCACCCTCACGATACACCTC |

*All positions are based on Morex v2
