# Supplementary material for: A novel locus conferring resistance to Puccinia hordei maps to the genomic region corresponding to Rph14 on barley chromosome 2HS
Source: Front Plant Sci. 2022 Oct 6;13:980870. doi: 10.3389/fpls.2022.980870 (PMC9583899; doi:10.3389/fpls.2022.980870)
Supplement: Supplementary file 2 [file Table_2.docx]

**Supplementary File Table S2:** Details of KASP markers designed to fine map *RphAGG396*.

| **Marker ID** | **Position*** | **SNP** | **Allele 1** | **Allele 2** | **Common primer** |
| --- | --- | --- | --- | --- | --- |
| HvGBSv2-902 | 35.26 | G/T | CCAGAAAAAGCCAGCTCAG | GCCAGAAAAAGCCAGCTCAT | CCAAAGGTGCATATCCGAAG |
| HvGBSv2-6994 | 37.2 | C/G | CACGGCGGAGATCGAC | CACGGCGGAGATCGAG | GAGAGTCCATGTCGGTCTCC |
| HvGBSv2-911 | 39.5 | A/G | GGGATGACGTCTCATGCTTA | GGGATGACGTCTCATGCTTG | ACCTCGTTTCTGTTGACGTG |
| HvGBSv2-7000 | 40.36 | C/T | GTGCAAATATTCTAGGTTCCATCC | GGTGCAAATATTCTAGGTTCCATCT | CAGGCCTTTCTCCTTGACTG |
| HvGBSv2-913 | 40.63 | G/T | CACTACCTACCAGAAGTTGGCG | ATCACTACCTACCAGAAGTTGGCT | TGCGTTTAGAGGGGATGAGT |
| HvGBSv2-919 | 42.57 | A/T | TGAGTTAACAGACTGAAAAAATTCCA | GATGAGTTAACAGACTGAAAAAATTCCT | ACCTGCTGTGTGTTGAATGG |
| HvGBSv2-920 | 42.99 | T/G | CGTACCTACGACACTAAACCCAGT | GTACCTACGACACTAAACCCAGG | CTTTGGCCGGAACTGAATAA |
| HvGBSv2-921 | 43.39 | G/A | CCACTGAAAGACCATGACACG | CCACTGAAAGACCATGACACA | ACACGAACGACAACACCAAG |
| HvGBSv2-923 | 44.06 | C/G | TGTGGCACCACCGAGC | TGTGGCACCACCGAGG | GGCCTATGCTCGTGAGGTT |
| HvGBSv2-926 | 45.35 | A/G | AAAGCATTACCAAATGCCACTA | AAGCATTACCAAATGCCACTG | TGCATGAAAGACAGGATTGG |
| HvGBSv2-7015 | 45.83 | T/G | CGAGAGCAAGAGAGGGGCT | AGAGCAAGAGAGGGGCG | TGTGGGCACGATGACTACAT |
| HvGBSv2-932 | 47.32 | T/C | GCAACACAAGCTCGCCT | GCAACACAAGCTCGCCC | TGCATGCATTGGAAGAAGAG |
| HvGBSv2-7022 | 48.26 | C/T | CAGCGACAACCAGCAGC | CCAGCGACAACCAGCAGT | TCCGTGTGGTTTTGTTGAAA |
| HvGBSv2-939 | 50.59 | C/A | TGGCAAGTGGGGGTGAC | GGCAAGTGGGGGTGAA | GAGTCCGAGTTCACCCAAAA |
| HvGBSv2-7031 | 51.28 | T/C | AAGCGCTCCCAGGAACAT | AAGCGCTCCCAGGAACAC | CTGGAGGAATTGGACGATGT |
| HvGBSv2-946 | 53.03 | C/T | CGGCGCTTCTCCTTATATTTC | CGGCGCTTCTCCTTATATTTT | ACCGCTCCAACGAAGATGTA |

* All positions are based on Morex v2
