# Supplementary material for: A novel locus conferring resistance to Puccinia hordei maps to the genomic region corresponding to Rph14 on barley chromosome 2HS
Source: Front Plant Sci. 2022 Oct 6;13:980870. doi: 10.3389/fpls.2022.980870 (PMC9583899; doi:10.3389/fpls.2022.980870)
Supplement: Supplementary file 3 [file Table_3.docx]

**Supplementary File Table S3:** Australian barley cultivars used for marker validation with marker MLoc-70.

| **Sample ID** | **Cultivar name** | **Sample ID** | **Cultivar name** |
| --- | --- | --- | --- |
| 1 | Arapiles | 36 | Litmus |
| 2 | Bandulla | 37 | Macquarie |
| 3 | Banks | 38 | Maltstar |
| 4 | Bass | 39 | Milby |
| 5 | Baudin | 40 | Moby |
| 6 | Beecher | 41 | Morrell |
| 7 | Binalong | 42 | Namoi |
| 8 | Bottler | 43 | Navigator |
| 9 | Brindabella | 44 | Pb216 |
| 10 | Charger | 45 | Prior |
| 11 | Chebec | 46 | RGT Planet |
| 12 | Clipper | 47 | Roe |
| 13 | Commander | 48 | Rosalind |
| 14 | Compass | 49 | Schooner |
| 15 | Cowabbie | 50 | Scope |
| 16 | Cutter | 51 | Shannon |
| 17 | Dhow | 52 | Skiff |
| 18 | Dictator | 53 | Skipper |
| 19 | Dictator 2 | 54 | Sloop |
| 20 | Doolup | 55 | Sloop SA |
| 21 | Empress | 56 | Sloop Vic |
| 22 | Fairview | 57 | Spartacus CL |
| 23 | Fathom | 58 | Stirling |
| 24 | Finniss | 59 | SY Rattler |
| 25 | Fitzgerald | 60 | Tallon |
| 26 | Fitzroy | 61 | Tilga |
| 27 | Flinders | 62 | Torrens |
| 28 | Franklin | 63 | Urambie |
| 29 | Gairdner | 64 | Vlamigh |
| 30 | Granger | 65 | Wimmera |
| 31 | Grout | 66 | Wyalong |
| 32 | Hamelin | 67 | Yambla |
| 33 | Hannan | 68 | Yerong |
| 34 | La Trobe | 69 | Flagship |
| 35 | Lindwall | 70 | ND24260 |
