# Supplementary material for: A novel locus conferring resistance to Puccinia hordei maps to the genomic region corresponding to Rph14 on barley chromosome 2HS
Source: Front Plant Sci. 2022 Oct 6;13:980870. doi: 10.3389/fpls.2022.980870 (PMC9583899; doi:10.3389/fpls.2022.980870)
Supplement: Supplementary file 4 [file Table_4.docx]

**Supplementary File Table S4:** Information for screening of AGG lines (AGG-396, AGG-397 and AGG-403) with INDELS and KASP markers

*
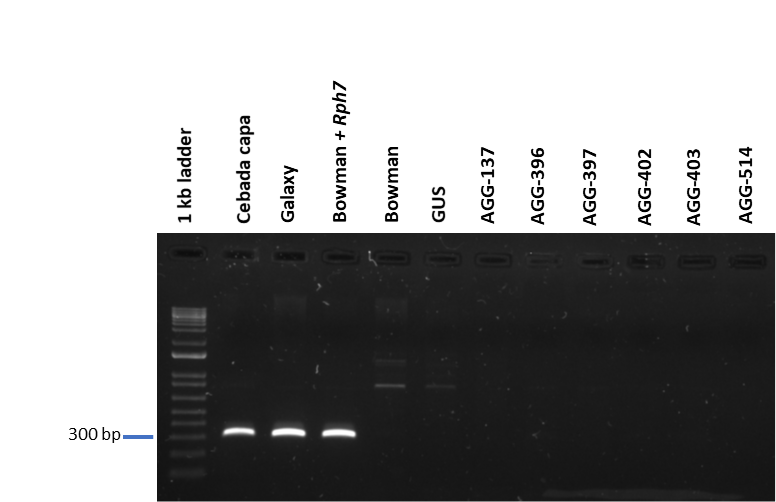
*

Figure 1. Gel results for screening of parental lines with markers linked to *Rph7*. Cebada Capa, Galaxy and Bowman+*Rph7* were used as positive controls while Bowman and Gus were used as negative controls for *Rph7.* A 300-base pair band can be seen for positive controls while negative controls along with six AGG-lines did not produce any band.


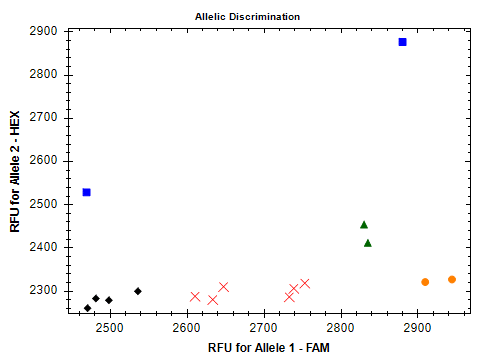


Figure 2. Screening of AGG lines with diagnostic KASP marker linked to *Rph15* (Chen *et al.* 2020). The AGG lines were detected negative for *Rph15*
